# Barriers and facilitators to disseminating quality improvement and patient safety research: a scoping review

**DOI:** 10.1093/intqhc/mzaf084

**Published:** 2025-08-28

**Authors:** Aisling Byrne, Roisin O’Malley, Paul O’Connor, Sinéad Lydon

**Affiliations:** Irish Centre for Applied Patient Safety and Simulation, School of Medicine, University of Galway, University Road, Galway, H91TK33, Ireland; Irish Centre for Applied Patient Safety and Simulation, School of Medicine, University of Galway, University Road, Galway, H91TK33, Ireland; Discipline of General Practice, School of Medicine, University of Galway, 1 Distillery Road, Newcastle, Galway, H91TK33, Ireland; Irish Centre for Applied Patient Safety and Simulation, School of Medicine, University of Galway, University Road, Galway, H91TK33, Ireland; Discipline of General Practice, School of Medicine, University of Galway, 1 Distillery Road, Newcastle, Galway, H91TK33, Ireland; Irish Centre for Applied Patient Safety and Simulation, School of Medicine, University of Galway, University Road, Galway, H91TK33, Ireland; Discipline of General Practice, School of Medicine, University of Galway, 1 Distillery Road, Newcastle, Galway, H91TK33, Ireland

**Keywords:** publishing, patient safety, quality improvement, improvement science, scholarly communication, authorship

## Abstract

**Background:**

Despite an abundance of quality improvement (QI) and patient safety (PS) research and on-the-ground initiatives, patients continue to suffer from iatrogenic harm. Addressing shortcomings in the dissemination of QI and PS research is one important step to improving patient care outcomes. The objective of this scoping review is to identify the barriers and facilitators, and related strategies/targets, to disseminating QI/PS research.

**Methods:**

The current review was conducted according to the JBI methodology for scoping reviews and PRISMA (preferred reporting items for systematic reviews and meta-analyses) extension for scoping reviews. A protocol was registered on the Open Science Framework website (doi: 10.17605/OSF.IO/RT57F). Databases searched included Medline, CINAHL, and Embase. Studies detailing barriers and facilitators, or solutions, to QI/PS research published between 2001 and March 2024 were included. Data on barriers and facilitators were coded deductively according to the theoretical domains framework (TDF).

**Results:**

Searches identified 5848 articles, of which 16 articles were included. Among studies seeking to *understand* (68.8%) barriers and facilitators to QI/PS dissemination, the TDF domain, environmental context and resources (ECR), was cited most frequently (68.8% of studies; e.g. availability of Standards for Quality Improvement Reporting Excellence (SQUIRE) guidelines), followed by skills (43.8%; e.g. poor reporting of QI/PS work), belief about consequences (37.5%; e.g. clearly highlighting the potential outcomes of dissemination) and goals (31.3%; e.g. early planning for dissemination). Studies seeking to address factors influencing dissemination (31.3% of studies) applied structured mentorship and curricular interventions to improve QI/PS dissemination, and suggested/enacted strategies were most commonly related to the individual’s ECR (25%; e.g. provision of a writing coach), behavioural regulation (25%; e.g. improved rates of publication), and knowledge (25%; e.g. workshop introducing QI tools).

**Conclusion:**

Organizational commitment and resourcing, access to QI/PS tools, programmes and reporting guidelines, and dedicated time, funding and resources are needed, alongside training programmes that target QI/PS knowledge and skills, and promotional pathways that nurture QI/PS activity. Research is required to cultivate effective QI/PS training programmes for qualified healthcare professionals, examine the identified factors in PS research specifically, and develop a consensus QI taxonomy to support the dissemination of QI research.

## Introduction

Quality improvement (QI) and patient safety (PS) research has demonstrated improvements across crucial patient outcomes, such as adverse drug events [1], central-line-associated bloodstream infections [2], surgical-site infections [3], and complications [3]. However, while there have been observed improvements in patient outcomes, continued deficits observed in PS, including high rates of iatrogenic harm [4–6], have prompted a focus on how such research is cultivated and spread.

Disseminating findings from QI/PS research is crucial in advancing the field and improving care [7]. Nonetheless, the dissemination of QI/PS research remains an enduring challenge to the advancement of this field. Research suggests that at least a quarter of clinical trials remain unpublished [8, 9] and between 25% and 60% of declined manuscripts never get published [10–12]. Further compounding this issue, is that much of the QI/PS research that is published is limited or deficient. For example, a report by the Lancet Global Health Commission found considerable heterogeneity in the methodological quality of QI/PS research, with a limited evidence base for many QI interventions [13, 14]. The potential benefits of QI/PS research for the organization, clinician, and patient have placed impetus on the need to better understand and support the dissemination of QI/PS research [13].

While a previous review [15] explored barriers and facilitators in the clinical audit literature, its scope was limited to clinical audit only. So, the full breadth of barriers and facilitators to reporting QI/PS research was not considered. Accordingly, the aim of our scoping review is to identify the barriers and facilitators, and related solutions, to disseminating QI/PS research. While the dissemination of research findings can take many forms, this study will focus on publications in journals and presentations at professional meetings [16], as they are two of the most common forms of disseminating research findings to the widest possible audience [16]. Further, presentations have the advantage of speed and direct interaction and journal publications are permanently available and more easily discoverable [16]. It is anticipated that fostering an understanding of this research literature will inform efforts to improve the quality and dissemination of research conducted in these areas, which in turn, will enable the successful implementation and sustainability of evidence into practice, ultimately leading to improved care delivery and outcomes for patients.

## Methods

### Study design

This scoping review was conducted in accordance with JBI guidance for scoping reviews [17] and the preferred reporting items for systematic reviews and meta-analyses extension for scoping reviews (PRISMA-ScR) [18]. A protocol using the JBI protocol template for scoping reviews was published on Open Science Framework website and updated in June 2025 [19]. Amendments have been made since the initial publication of this protocol. Studies focused on the conduct of research only (and not dissemination) were ultimately excluded, while those focused on addressing barriers and facilitators to QI/PS research dissemination (e.g. interventions/tools to improve publication) were included. Further, duplicate papers were identified in Microsoft Excel© without the use of EndNote.

### Search strategy

Searches were conducted across Medline (Ovid), CINAHL (EBSCO), and Embase (Ovid) in April 2024. Development of the search strategy was informed by similar reviews [20, 21] and was reviewed by a research librarian at the authors’ university. The search strategy included keywords and MeSH terms and was adapted for each database (see Supplementary Material S1 for Medline search strategy). Due to a lack of clarity in the wording of known relevant studies, reference list reviews were also conducted as they are useful when there is difficulty identifying all papers through hand-searching and database searching alone [22]. Searches were limited to the English language.

### Study selection

#### Inclusion and exclusion criteria

To be included, studies were required to: focus on the identification or amelioration of barriers and/or facilitators to disseminating QI/PS research; involve/target healthcare workers, managers or researchers in any healthcare setting; and be published in English. The QI field is generally considered to encompass QI research (focused on the production of generalizable scientific knowledge applicable across care settings) and QI activities/projects (typically focused on generating knowledge for local improvement in specific settings) [23]. While research on either type of QI were eligible for inclusion, the study had to focus on dissemination through journal publications or presentations, and so studies focused on developing QI reports only were excluded. Further, only studies published since 2001 were included due to the increase in PS research following the 1999 IOM report ‘To Err Is Human’ [24]. Finally, any study design was considered, including original (e.g. qualitative, quantitative) and nonoriginal (e.g. editorial, commentaries) studies.

Studies were excluded if they focused on: topics other than barriers, facilitators, and/or suggested strategies to disseminating QI/PS research; nonhealthcare research or researchers working outside of healthcare; clinical audit or safety in clinical trials, or quality broadly, rather than QI/PS research specifically; barriers/facilitators associated with certain research or specific QI projects; a single barrier/facilitator to research; dissemination/implementation of QI/PS research into practice, rather than the dissemination in terms of publication and/or presentation; improving QI/PS knowledge only, as opposed to the dissemination of QI/PS research; or reporting quantitative data on the conduct/dissemination of research only without actively seeking improvement. Studies published before 2001 or in a language other than English were excluded.

### Screening

Titles and abstracts for all returns were screened by A.B., who retained any studies that appeared eligible or where eligibility was unclear. Full-text screening and reference list review were conducted by R.O.M. During both the initial database screening and full-text and reference list reviews, the full research team met regularly to discuss any uncertainties regarding the eligibility of certain papers that arose during screening, which were resolved through discussion as a team. Duplicate papers were identified during initial database screening in Microsoft Excel©.

### Data extraction

A data extraction form was drafted (see Supplementary Material S2) as per guidance from Pollock *et al.* [25], to extract data on study author, year of publication, study type (original, nonoriginal), methodology, study aim, participants, setting, and findings (barriers and facilitators, and/or [suggested or enacted] intervention/study targets). Intervention/study targets included the strategies employed (e.g. provision of mentor) or areas targeted (e.g. increases in QI knowledge) by studies that addressed factors influencing QI/PS dissemination. The data charting form was piloted on 25% of studies by A.B. and R.O.M. in June 2024 and modified as necessary. Remaining data extraction was carried out by A.B. and R.O.M. independently. Any disagreements that arose were resolved through discussion.

### Data synthesis

As per JBI guidance for scoping reviews [17], data were synthesized using two methods; first, a descriptive numerical summary for demographic study information was developed. Second, a narrative synthesis of study outcomes was developed, as per guidance by Popay *et al.* [26]. Tables and figures were utilized to summarize demographic information and numerical data as deemed useful.

Codes were also applied deductively to the extracted data (see Table 1 for coding procedures). The study aim was coded as either understanding or addressing barriers and facilitators to QI/PS research. Second, a deductive content analysis approach was used to code extracted ‘findings’ data according to the theoretical domains framework (TDF) [27]. The TDF is a theoretical framework encompassing 14 social and structural determinants of behaviour—originally developed to identify influences on health professional behaviour when implementing evidence-based recommendations [28]. This framework is commonly applied in systematic reviews to understand barriers and facilitators influencing healthcare providers’ behaviour [29, 30] and inform subsequent intervention/change efforts [31, 32].

**Table 1.** Procedure for deductive coding of extracted data

Category	Definition
**Study aim**	
**Understanding barriers and facilitators**	Studies which sought to determine barriers and facilitators, or which provided commentary on personal expertise on barriers and facilitators to QI/PS research
**Addressing barriers and facilitators**	Studies which developed and piloted or implemented a tool or intervention to support the dissemination of QI/PS research
**Understanding and addressing barriers and facilitators to disseminating QI/PS research**	
**Theoretical domains framework, according to Michie *et al.* [27]** Barriers and facilitators identified within studies were coded according to the TDF domains. The enacted/suggested target or strategies extracted from studies *addressing* barriers and facilitators—commonly interventional studies—were also coded according to the TDF domains (e.g. educational intervention targeting changes in QI/PS knowledge coded as the TDF domain, Knowledge).	
**Knowledge**	An awareness of the existence of something
**Skills**	An ability or proficiency acquired through practice
**Social/professional role and identity**	A coherent set of behaviours and displayed personal qualities of an individual in a social or work setting
**Beliefs about capabilities**	Acceptance of the truth, reality or validity about an ability, talent or facility that a person can put to constructive use
**Optimism**	The confidence that things will happen for the best or that desired goals will be attained
**Beliefs about consequences**	Acceptance of the truth, reality, or validity about outcomes of a behaviour in a given situation
**Reinforcement**	Increasing the probability of a response by arranging a dependent relationship, or contingency, between the response and a given stimulus
**Intentions**	A conscious decision to perform a behaviour or a resolve to act in a certain way
**Goals**	Mental representations of outcomes or end states that an individual wants to achieve
**Memory, attention, and decision processes**	The ability to retain information, focus selectively on aspects of the environment and choose between two or more alternatives
**Environmental context and resources**	Any circumstance of a person’s situation or environment that discourages or encourages the development of skills and abilities, independence, social competence and adaptive behaviour
**Social influences**	Those interpersonal processes that can cause individuals to change their thoughts, feelings or behaviours
**Emotion**	A complex reaction pattern, involving experiential, behavioural, and physiological elements, by which the individual attempts to deal with a personally significant matter or event
**Behavioural regulation**	Anything aimed at managing or changing objectively observed or measured actions

## Results

**Figure 1:**
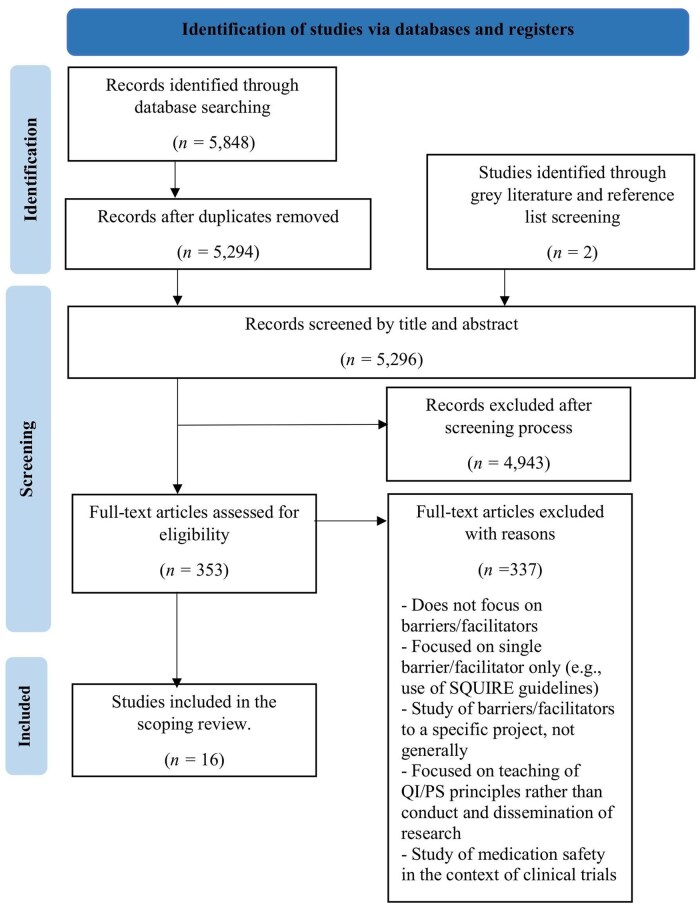
PRISMA-ScR flow chart detailing study selection process

Database searches identified 5848 articles (Fig. 1); 353 underwent full-text review. Two papers were identified during reference list screening, resulting in 16 articles meeting inclusion criteria [33–48]. Detailed study-by-study data are provided in Supplementary Material S2, while Table 2 offers an overview of study characteristics.

**Table 2.** Summary of the characteristics of included studies (*N = *16)

Characteristics	Studies *N* (% of all studies)
Year of publication	
2001–05	2 (12.5)
2006–10	0 (0)
2011–15	7 (43.8)
2016–20	4 (25)
2021–24	3 (18.8)
Study type	
Original	8 (50)
Interventional—curriculum implementation	1 (6.3)
Interventional—structured research programme	3 (18.8)
QI tool development	1 (6.3)
Qualitative inquiry	2 (12.5)
Descriptive survey	1 (6.3)
Nonoriginal***—***Commentary	8 (50)
Study aim	
Understand barriers/facilitators to QI/PS research	11 (68.8)
Address barriers/facilitators to QI/PS research	5 (31.3)
Country	
USA	9 (56.3)
UK	2 (12.5)
Canada	2 (12.5)
Australia	1 (6.3)
Multicountry (Australia, Europe, North America)	1 (6.3)
Participants^a^	
Qualified healthcare professionals	9 (56.3)
Researchers/academics	4 (25)
Postgraduate trainees/residents	3 (18.8)
Healthcare students	1 (6.3)
Experts/specialists in QI/PS	3 (18.8)
Journal editors/administrators	2 (12.5)
Undefined	3 (18.8)

a Studies do not total 100% as some studies addressed multiple categories.

### Exploring the barriers, facilitators, and intervention targets identified across included studies

Across the 16 included studies, 81 facilitators, 57 barriers, and 41 intervention targets (*N = *179) were identified and subsequently coded according to TDF domains.

### Environmental context and resources (*N* = 15 studies; 93.8%)

Table 3 provides an overview of the barriers, facilitators, and intervention/study targets. With regard to understanding factors influencing dissemination, most barriers (50%; e.g. fitting QI into traditional academic publishing [48]) and facilitators (62.5%; Standards for Quality Improvement Reporting Excellence (SQUIRE) guidelines [44]) were identified in the environmental context and resources (ECR). The included research also considered how ECR could be targeted within initiatives to address difficulties in disseminating QI work (25%), which included the provision of writing workshops with an external writing coach [40].

**Table 3.** Summary of barriers, facilitators, and interventional/study targets across studies (*N = *16)

Barriers (*N = *9 studies)	*N* (%)	Facilitators (*N = *10 studies)	*N* (%)	Intervention target (*N = *5 studies)	*N* (%)
Environmental context and resources	8 (50)		10 (62.5)		4 (25)
*Reporting and writing up QI/PS*					
- Fitting QI/PS work into traditional publication guidelines	3 (18.8)	- Use of SQUIRE guidelines to support the reporting of QI/PS work	7 (43.8)	- Linking the manuscript outline with the structure of an existing quality framework eased transition to the manuscript format [40].	1 (6.3)
- Inconsistent and variable application of terminology across disciplines	1 (6.3)	- Use of existing models/tools (e.g. TIDieR checklist, MUSIQ tool) to improve the reporting of study	2 (12.5)		
		- Adherence to journal guidelines and professional standards in the area	2 (12.5)		
		- Teach key elements of QI/PS and its write-up in medical school	1 (6.3)		
*National resources and guidance for conducting and publishing QI/PS research*					
- Broad scope of QI causes uncertainty about where QI work should be published	1 (6.3)	- Availability of journals and databases dedicated to QI, and/or a QI section in discipline-specific journals	1 (6.3)	- Use of a dissemination planning tool provided a useful structure to support dissemination [33].	1 (6.3)
- Data may be difficult to access, and available data may not be rigorous	1 (6.3)	- Availability of validated and easy to use forms of data collection	2 (12.5)	- Use of quality journal (e.g. BMJ quality) as a resource was useful [42].	1 (6.3)
		- Access to national QI programmes, resources, and tools to aid with conduct, reporting and publishing of QI/PS	2 (12.5)		
*Organizational infrastructure to support QI/PS work*					
- Difficulties finding faculty trained in QI/PS	2 (12.5)	- Reward and nurture QI/PS work at an organizational level through promotions, mentoring, training	2 (12.5)	- Facilitate the logistics of training sessions (e.g. parking, alternating session times, food) [40].	1 (6.3)
		- Develop fellowships and training programs in QI/PS	3 (18.8)	- Mentoring for QI projects and writing group workshops with external writing course [39, 40, 42].	3 (18.8)
		- Develop and embed QI research units and/or forums	2 (12.5)	- Multifaceted curricular intervention implemented to increase student scholarly output including abstract and poster workshops [35].	1 (6.3)
		- Organizational leader/head aware of planned dissemination activities	1 (6.3)		
*Access to funding and resourcing to support writing and publishing*					
- Limited funding available to conduct and publish QI/PS	2 (12.5)	- Develop a small organizational grant/fund to support QI manuscript preparation and publishing	1 (6.3)		
		- Review and connect teams/individuals to available funding opportunities and resources	2 (12.5)		
		- Leverage existing institutional resources or grants (e.g. recruiting ‘free’ students and trainees)	1 (6.3)		
*Time needed to conduct, write-up and publish QI/PS work*					
- Limited time to conduct/write-up research due to clinical commitments	5 (31.3)	- Develop the most rigorous study design possible without disrupting normal work unduly	1 (6.3)	Mentors reported spending ‘a reasonable amount of time’ on each activity related to mentoring [39].	1 (6.3)
		- Ensure protected time for QI work	1 (6.3)	Dedicated time during the workshop needs to be set aside for actual writing by the team members [40].	1 (6.3)
*Ethical and legal considerations*					
- Potential for legal and ethical issues to arise during the conduct and publishing of QI/PS work	1 (6.3)	- Ensure publication/presentation complies with ethical/legal standards	1 (6.3)		
- Ethical approval may be required to publish in journal, even if not required to conduct the study	1 (6.3)	- Carefully consider a study’s ethics and work with IRBs to determine whether IRB approval needed	2 (12.5)		
Skills	6 (37.5)		5 (31.3)		3 (18.8)
- Poor quality and completeness in reporting and presenting QI/PS (e.g. lack of consideration of context or theory)	4 (25)	- Consider theory and context when reporting QI/PS work and report findings using appropriate wording and supported by useful graphs	3 (18.8)	- Writing team members need technical assistance with drafting and editing skills [40].	1 (6.3)
- Lack of experience or training in QI/PS theory or conducting/reporting research	3 (18.8)	- Maintain QI/PS journal and track study progress (e.g. QI diary to manage contextual information)	1 (6.3)	- Improving skills in research and QI method. The number agreeing that they understand the statistics that they encounter significantly increased [39]. Majority agreed that training had been effective in teaching them how to perform QI project and changed how they would perform QI in the future (10/14) [42].	1 (6.3)
- Poor quality or in-appropriate research methods selected or undertaken as part of a study (e.g. small sample sizes)	2 (12.5)	- Use rigorous and appropriate research methods to address the study’s research aim	3 (18.8)		
		- Use additional tools to provide relevant information (e.g. online supplementary materials, podcasts)	1 (6.3)		
Knowledge	5 (31.3)		2 (12.5)		4 (25)
- Lack of awareness of relevant journal guidelines and unfamiliarity with QI journals	3 (18.8)	- Familiarize self with relevant guidelines and identify suitable journals	1 (6.3)	- One manuscript rejected by the journal—need more rigorous selection of projects [40].	1 (6.3)
- Unfamiliarity with QI methodologies and tools, and the difference between QI and QI research	2 (12.5)	- Select a quality problem with gaps in knowledge of effective interventions	1 (6.3)	- Dissemination tool improved participant knowledge of the dissemination process and where research should be applied [33].	1 (6.3)
- Unfamiliarity in the key reporting elements of QI work	1 (6.3)			- Workshop and mentorship programme targeted knowledge (e.g. introducing QI tools, discussion of previous QI projects). While an increase in QI knowledge was observed (46% vs 54%) it was not significant (*P* = .17) [42]. Another study found no changes in students’ knowledge following a mentorship programme [39].	2 (12.5)
- Lack of understanding around which quality problems should be studied and/or represent ‘new knowledge’	1 (6.3)				
Beliefs about consequences	4 (25)		5 (31.3)		3 (18.8)
- Belief that project not suitable for publishing due to scale or view that QI less valuable than HSR	3 (18.8)	- Select QI/PS projects that bring value to patients and the quality and safety of healthcare	2 (12.5)	- 73% felt their project would make them more competitive for a job or residency [39].	1 (6.3)
- Reporting bias and perception that only positive findings will be published	2 (12.5)			- Teach the value of research an importance of its dissemination. Researchers should understand the dissemination process, recognizing the importance of research’s use and its practical application [31].	1 (6.3)
- Individual resistance to a QI/PS project during implementation or dissemination	1 (6.3)	- Carefully and clearly highlight all the potential benefits, goals, and outcomes of project to stakeholders	3 (18.8)		
Goals	2 (12.5)		5 (31.3)		2 (12.5)
- Lack of planning, including publication goals, at the start of a project	1 (6.3)	- Early assessment of goals and dissemination plan, including the selection of a suitable journal	4 (6.2)	- Early planning. Poor preparation and planning at initial project design stage [42].	1 (6.3)
- Goals of the study are not clear or have not been explicitly defined	1 (6.3)	- Set explicit, actionable and measurable goals for the project with clear timelines	3 (18.8)	- Having a defined submission date. Setting an expectation that teams would meet the external deadlines imposed by the journal editor [40].	1 (6.3)
		- Ensure accountability, set clear roles, and discuss authorship early in the project	4 (25)		
Social influences	1 (6.3)		4 (25)		3 (18.8)
- Potential for QI/PS research, and its dissemination, to marginalize people	1 (6.3)	- Use influence to encourage best practice in publishing (e.g. encourage stakeholders to use SQUIRE guidelines)	2 (12.5)	- Feedback from mentor on QI proposal and planning helpful [42]. Participants appreciated peer support, timely coaching and support from workshop faculty [40].	1 (6.3)
		- Collaborate with and get input from other colleagues	2 (12.5)	- Participants plan to use the dissemination tool amongst the research team to gather additional input [33].	1 (6.3)
		- Be creative in recruiting others to support the study	1 (6.3)	- Commitment to person known and respected–journal editor present at workshop [40]. Limited project buy-in from colleagues [42].	2 (12.5)
Behavioural regulation	1 (6.3)		1 (6.3)		4 (25)
- Teams/individuals start writing too late	1 (6.3)	- Begin writing the manuscript during the QI/PS project	1 (6.3)	- Most students (82%–97%) completed their projects by the set publication date [39, 40].	2 (12.5)
				- Four studies had their projects accepted (17%–92% of projects) for publication [35, 39, 40, 42]; a 28.5% increase from the previous year [35].	3 (18.8)
				- Many presented their work (49%–58% of projects) [35, 39]; a 54% increase in one study [35].	2 (12.5)
Reinforcement	2 (12.5)		3 (18.8)		1 (6.3)
- Standard promotions paradigms may not adequately reward individuals their scholarly and academic	2 (12.5)	- Scholarly work is rewarded and nurtured	2 (12.5)	- 88% of mentors somewhat or extremely satisfied with the final project, 73% thought the students’ work was beneficial to the mentor [39].	1 (6.3)
		- Acknowledge all those who contributed to project	1 (6.3)		
Belief about capabilities	1 (6.3)				1 (6.3)
- Not acknowledging or discussing limitations of the study	1 (6.3)			- Students’ confidence in their ability to understand and participate in research increased (*P* < .001) [39]. Confidence in QI knowledge and skills improved after training, maintained at 4 months [42].	1 (6.3)
Intentions			1 (6.3)		1 (6.3)
		- Personal or organizational interest may encourage publishing	1 (6.3)	- No significant changes in students’ attitudes, however, interest in learning about biostatistics decreased slightly [39].	1 (6.3)
Social/professional role identity	1 (6.3)				
- Individuals not having an academic focus	1 (6.3)				
Emotion	1 (6.3)				
- QI may cause pressure or anxiety for those involved	1 (6.3)				

### Skills (*N* = 10; 62.5%)

Across studies that sought to understand factors associated with QI/PS dissemination, several barriers (37.5%; e.g. difficulties reporting active ingredients and contexts in QI [37]) and facilitators (31.3%; e.g. detail the most relevant contextual factors (staffing, clinic set-up, etc.) [45]) were related to skill. In studies that addressed skill-related factors (18.8%), providing technical assistance with drafting and editing manuscripts [46] was suggested as one strategy.

### Knowledge (*N* = 9; 56.3%)

Studies seeking to understand barriers and facilitators to dissemination identified many knowledge-related barriers (31.3%; e.g. finding journals that understand and publish QI [41]) and facilitators (12.5%; e.g. being familiar with relevant guidelines and journals [41]). Providing a workshop where teams are introduced to QI tools and projects, including the critical analysis of a published report [42], was one strategy enacted to address knowledge and improve QI/PS dissemination (25%).

### Beliefs about consequences (*N* = 9; 56.3%)

Several barriers (25%; e.g. individual staff members may be resistant to a QI project, and the potential for the research and it’s write-up to focus on specific groups or individuals [43]) and facilitators (31.3%; e.g. the use of thoughtful language to explain goals/findings of research, clearly indicating that it is targeting a system or process [43]) related to beliefs about consequences were identified in studies seeking to understand factors influencing QI/PS dissemination. In studies that addressed such factors (18.8%), completing a QI project and exposure to the full research process made residents feel more competitive for a job/residency [39].

### Goals (*N* = 7; 43.8%)

With regard to understanding factors influencing dissemination, goals could function as a barrier (12.5%; e.g. inexplicit initial assessment of publication goals [41]) or a facilitator (31.3%, e.g. set clear, measurable aims, and timelines [36]). To address barriers and facilitators (12.5%), setting an expectation that teams meet the external publication deadlines imposed by the journal editor [40] was one effective strategy detailed relating to goals.

### Social influences (*N* = 7; 43.8%)

As seen in Table 3, both barriers (6.3%; e.g. potential for presentation to marginalize certain individuals/groups [34]) and facilitators (25%; e.g. getting input from experienced colleagues on proposed presentation [34]) related to social influences were identified in studies seeking to understand QI/PS dissemination. In studies that targeted social influences to address difficulties in dissemination (18.8%), the provision of peer support, timely coaching, and faculty support to assist with the publication of QI projects [40] was considered an effective strategy.

### Behavioural regulation (*N* = 6; 37.5%)

With regards to understanding factors influencing dissemination, behavioural regulation could function as both a barrier (6.3%; e.g. starting to write too late [41]) or facilitator (6.3%; e.g. begin to write the paper during the conduct of the QI project [44]). To address barriers and facilitators to dissemination (6.3%), providing a multifaceted educational intervention proved an effective strategy in improving publication rates [35].

### Reinforcement (N = 5; 31.3%)

With regards to understanding factors influencing dissemination, both barriers (12.5%; e.g. lack of incentive for publishing findings [47]) and facilitators (18.8%; e.g. rewarding scientific inquiry through promotions [46]) related to reinforcement were identified. One study that addressed such factors (6.3%) found that mentors perceived the students’ work as beneficial to the mentor [39].

### Beliefs about capabilities (*N* = 2; 12.5%)

In efforts to understand factors influencing dissemination, the failure to discuss limitations was identified as a barrier (6.3%) related to beliefs about capabilities. To address challenges in disseminating (6.3%), the provision of a workshop and mentoring programme that supported QI project publication was found to improve confidence in QI knowledge and skills [42].

### Intentions (*N *= 2; 12.5%)

With regard to understanding factors influencing dissemination, one facilitator related to intentions (6.3%; e.g. personal/organizational self-interest might prompt publication [37]) was identified. In one study that sought to address dissemination (6.3%), students’ interest in learning about biostatistics decreased slightly after training and completion of a research project [39].

### Social/professional role identity (*N* = 1; 6.3%)

As seen in Table 3, one barrier related to an individual’s social/professional role identity (6.3%; e.g. having a nonacademic hindered publication [38]) was identified in efforts to understand factors influencing dissemination.

### Emotion (*N* = 1; 6.3%)

In efforts to understand dissemination, the potential for QI endeavours to cause anxiety or pressure [43] was identified as an emotion-related barrier (6.3%).

## Discussion

### Statement of main findings

This review collated research literature which sought to understand and address barriers and facilitators to disseminating QI/PS research. Organizational commitment and resourcing, access to QI/PS tools, programmes and reporting guidelines, and dedicated time, funding and resources are needed to support QI/PS dissemination, alongside training programmes and education that target QI/PS knowledge and skills, and promotional pathways that nurture QI/PS activity in practice. Healthcare professionals looking to disseminate their QI/PS work should pick projects that matter to patients, set explicit goals and timelines early in the project, and clearly communicate the value of publishing and expected outcomes of disseminating QI/PS work.

### Strengths and limitations

A broad list of search terms were developed alongside reference list searches in order to increase the comprehensiveness of the searches and ensure that all relevant research were identified. Nonetheless, development of the search strategy was challenged by heterogeneity and inconsistency in reporting of QI initiatives [49], and so, relevant papers may have been missed due to the inconsistent terminology used across studies. A grey literature search was not performed as, although it may broaden the scope, there are recognized methodological challenges in interpreting grey literature [50]. Our search was limited to English-language papers, which can be associated with bias and reduced generalizability [51]; however, previous studies have found little impact of limiting reviews to English-language papers only [52]. Finally, critical appraisal of included studies was not performed, as it is not deemed mandatory in a scoping review of this nature [17]. With regards to the analysis, the level at which studies explored barriers and facilitators varied, with some looking at the organizational level [46], while others looked at the team [41] or individual [37, 38]. While this made coding somewhat challenging, it allowed for a greater variety of multilevel system factors to be identified, and at least two authors coded each factor to add credibility to the analysis.

### Interpretation within the context of the wider literature

Environmental context and resource factors were among the most common barriers and facilitators identified. Many of these extrinsic factors are well-established as influencing the publication of research more broadly, including time [53–56], funding [54, 57], selection of research methods and measures [55], and organizational research infrastructure [57]. These factors were also identified as important in the clinical audit literature [15]. Organizations can provide targeted support to address such challenges to scholarly productivity [56]. However, the nature of the barriers reported may suggest that even in a resource-rich setting, academics may perceive barriers to disseminating QI/PS work [56]. For example, specific to QI/PS research, challenges reporting QI/PS work and identifying suitable journals were noted. Standardizing the reporting of adaptive and iterative projects leads many QI projects to go unpublished [48], and has prompted many studies, including those synthesized herein, to advocate for the use of SQUIRE guidelines [58] to support QI/PS reporting. Nonetheless, a recent analysis of 160 QI projects found that considerable reporting inadequacies remain [59], suggesting the need for greater uptake of QI formatting guidelines. It is possible that these distal influences identified herein underlie many of the more proximal challenges reported (e.g. poor reporting of QI/PS work) [37]. While these factors can be more challenging to shift, it is pertinent that future training programmes and interventions target the environmental context in addition to the healthcare professional’s behaviour.

Knowledge and skills accounted for almost one-fifth of the barriers and facilitators identified. A lack of experience and skills in formatting, organizing, and writing up manuscripts is considered a primary barrier to research dissemination across fields [54, 56, 57], and a top training priority for academic clinicians [56, 60]. However, it is possible that difficulties with writing scientific manuscripts are further compounded by nuances in QI/PS reporting. For example, many studies cited challenges to reporting contextual and theoretical information key to the study. Research suggests securing improvements in reporting could yield more widespread improvements in care [37].

Research education remains a key strategy to build research capacity for those working in healthcare settings [61]. Developing training programmes to target QI/PS knowledge and skills may help to improve the quality of QI dissemination [45]. The four interventional studies in this review generated improvements in skills [39, 42], and rates of publication [35, 39, 40, 42] and presentation of findings [35, 39]. Previous reviews of research curricula have also found that mentorship, research training, protected time, and a supportive environment improve research output among residents [62] and medical students [63]. Given that interventional studies mostly focused on trainees and medical students, the development and evaluation of evidence-based QI and PS training for qualified healthcare professionals are warranted.

Psychosocial domains, such as goals, beliefs about consequences, social influences, and reinforcement were frequently cited in our review. Again, many of these factors have been identified as barriers to publishing research broadly, including being motivated due to career progression [53], belief in the value of publishing [53, 57], interest in conducting/disseminating research [53], mentorship [57], and sufficient planning [15]. The importance of such psychosocial factors echoes Dixon-Woods’ suggestion of drawing on ‘softer’ incentives for staff, such as intrinsic motivation and feedback of outcomes, as well as ‘harder’ incentives such as progressing continuous professional development [64]. For academics experiencing psychological or emotional barriers to QI dissemination, support groups for academic writing have been recommended, to help foster a positive attitude about writing, establish collaborative relationships, and facilitate dissemination outputs [56, 65, 66]. Despite representing many of the barriers and facilitators identified, these psychosocial factors were seldom addressed by training interventions in this review. Thus, research efforts to develop effective training programmes in QI/PS need to ensure that psychosocial influences are also addressed.

### Implications for policy, practice, and research

A number of further considerations for future research can be suggested. As found by previous studies [59, 67], search strategy development was challenging due to lack of a defined QI terminology and inconsistencies in QI reporting. We recommend that a consensus QI taxonomy is developed, in order to enhance dissemination and improve completeness of future reviews. Moreover, only one study in the review targeted PS. As fields, QI and PS share similarities, but ultimately, have distinct theoretical and practical underpinnings [68]. As such, it is vitally important that future research explores barriers and facilitators to PS research specifically, to confirm whether similar factors impede/facilitate dissemination in both fields.

Organizations should seek to cultivate the facilitators identified by the review and the impact of this on dissemination activity. For example, developing small grants programs may allow QI teams time to work on their manuscripts [41], providing services to support scholarly activity (e.g. manuscript editing, statistical analyses) can enable better dissemination [56], and rewarding QI involvement may encourage scholarly activity in practice [38, 41].

Quality improvement should be integrated into continuing medical education for clinicians to enhance awareness and share examples of successful dissemination [38]. In addition to organizational efforts, greater inclusion of QI methodology in resident and fellowship training programmes is recommended [38]. In the context of university-affiliated organizations, recognition and academic promotion for QI dissemination are needed alongside opportunities for capacity building [38, 69]. As a field, QI custodians need to work with researchers and consumers to develop more appropriate approaches that address enduring challenges to effective QI/PS dissemination [37].

Finally, it is important to note that while the dissemination of QI/PS findings is a critical first step in optimizing the impact of QI and PS work, researchers and clinicians should not expect that practice change will occur once research findings are published [16]. Instead, the effective dissemination of QI/PS work must be followed by the active implementation of findings and the sustained integration of innovations if the ‘knowledge-to-action’ gap is to be addressed and meaningful changes in care delivery and patient outcomes are to be achieved [70].

## Conclusion

Improving the safety and quality of care patients receive will require well-designed and executed QI and PS projects founded on a foundation of well-conducted and reported QI/PS research. There is a need to cultivate adequate QI/PS training efforts, explore findings further in PS specifically, and develop a consensus QI taxonomy to advance the dissemination and utilization of QI/PS research further.

## Supplementary Material

mzaf084_Supplementary_Data

## Data Availability

The data underlying this article are available in the article and in its online supplementary material.
