# Supplementary material for: Barriers and facilitators to disseminating quality improvement and patient safety research: a scoping review
Source: Int J Qual Health Care. 2025 Aug 28;37(3):mzaf084. doi: 10.1093/intqhc/mzaf084 (PMC12459987; doi:10.1093/intqhc/mzaf084)
Supplement: mzaf084_Supplementary_Data [file mzaf084_supplementary_data.zip › Supplementary material 2.docx]

**Supplementary Material 2**. Data charting table

| 1. **STUDIES ON UNDERSTANDING BARRIERS & FACILITATORS** | | | | | |
| --- | --- | --- | --- | --- | --- |
| **Author; Year** | **Study Type** | **Methodology** | **Aim** | **Participants & Setting** | **Outcomes** |
| Cleary, Hunt, Walter et al (2003) | **Non-original** | **Commentary**  This research note provides protocols and a checklist that may be relevant for clinicians and researchers to review and appraise their work before oral or written presentation | **Understanding barriers and facilitators**  The purpose of this research note is to encourage discussion and propose guidelines for disseminating findings relating to quality and research projects | **Mental health clinicians and researchers**  **Mental health care**  **Australia**  Mental Health Service clinicians and researchers | Facilitators:   - - **Social influences**: Get input. Have experienced colleagues review the presentation   - **Social influences**: Ensure the presentation does not marginalize or discriminate against specific individuals or groups.   - **Environmental Context & Resources**: Ensure the presentation complies with ethical standards/privacy and confidentiality.   - **Environmental Context & Resources:** Ensure the presentation complies with professional and organisational standards   - **Reinforcement:** Ensure acknowledgement been given to all who provided assistance, and disclosure of funding any benefits received acknowledged.   - **Environmental Context & Resources:** Ensure department head/relevant personnel have been informed of the presentation and sent a copy.   - **Goals:** Ensure the presentation meets Joint NHMRC/AVCC guidelines in regard to authorship.   Barriers:   - - **Environmental Context & Resources:** The potential for legal problems to arise (sensitive info/confidentiality)   - **Social influences:** The potential for the presentation to marginalise or discriminate against individuals/groups |
| Goldmann (2011) | **Non-original** | **Commentary**  This paper provides 10 tips to increase the rigour and credibility of quality improvement work regardless of where it is published, taught or posted. | **Understanding barriers and facilitators**  This paper provides personal advice (10 ‘tips’) for choosing, designing, implementing and publishing work that improves patient care and advances the field | **Healthcare providers**  **Setting not defined**  **USA**  No subjects/participants were described, however, the article was written for ‘healthcare providers’ | Facilitators:   - **Beliefs about consequences:** Select projects that make a major difference to the patients and healthcare providers who will participate in them - **Goals**: Set bold, clear, measurable aims and a timeline for achieving them - **Goals**: Assemble a multidisciplinary team tailored to the aim of the project, with clear roles and authorship - **Social influences**: Be creative in recruiting experts as investigators - **Environmental Context & Resources:** Develop the most rigorous study design possible without disrupting normal work unduly – incorporate data collection into the usual activities of professional staff - **Environmental Context & Resources:** Do everything possible not to sacrifice data quality and completeness – avoid intermittent/non-standardised data collection, use easy-to-use data collection forms/instruments - **Reinforcement**: Take advantage of emerging certification requirements for clinical staff, and make improvement academically viable in institutions where promotions matter - **Environmental Context & Resources:** Do not assume that major external funding is necessary to perform credible improvement work – leverage existing institutional resources or small grants. Look for “free” minds and hands e.g. university graduate students/post-docs - **Environmental Context & Resources:** Pay careful attention to the ethics of all QI work, but craft projects that are unlikely to require formal IRB approval – QI depts should work with their IRBs to develop a consensus on which projects don’t need IRB approval (minimal risk, compliant with relevant acts and other patient protection standards) - **Environmental Context & Resources:** use Standards for Quality Improvement Reporting Excellence (SQUIRE) reporting guidelines - **Goals**: Whenever possible, anticipate possible publication – Write an abstract while designing the study |
| Jones, Dixon-Woods, Martin (2019) | **Original** | **Qualitative study**  Semi-structured interviews to explore why reporting of QI interventions and QI methods might pose challenges | **Understanding barriers and facilitators**  **Research**  We aimed to identify the influences on reporting QI in the area of perioperative care, with a view to informing improvements in reporting QI across healthcare | **Stakeholders involved in or influencing the publication, writing or consumption of reports of QI studies**  **Perioperative care, healthcare and academic organisations**  **Six countries across North**  **America, Australia and Europe**  42 stakeholders involved in or influencing the publication, writing or consumption of reports of QI studies in perioperative care, in healthcare and academic organisations in Australia, Europe and North America  Participants from 6 countries, including 15 authors of QI reports, 12 consumers of QI reports (practitioners who apply QI research in practice), 11 journal editors, 4 authors of reporting guidelines | Facilitators   - **Environmental Context & Resources**: having journals dedicated to QI, and/or a QI section in surgical journals - **Social influences**: encouraging all QI stakeholders to use the SQUIRE guidelines including journal editors and peer reviewers - **Environmental Context & Resources:** having a central database of QI work in surgery - **Environmental Context & Resources**  wider use of the MUSIQ tool, extending the MUSIQ tool to highlight contextual features known to affect QI in surgery - **Environmental Context & Resources**: including the study of context in medical school curricula on QI - **Skills:** using terms such as ‘portable’ and ‘reproduce’ in lieu of ‘generalisable’ and ‘replicate’ to encourage understanding that not all mechanisms can be replicated exactly and some need to be adapted to other settings - **Skills**: managing contextual information using objective scales; QI diaries kept by the researchers, which participants likened to lab books; external independent evaluation; and ethnography - **Intentions:** Personal or organisational self-interest might prompt QI authors to seek to publish their work - **Belief about consequences**: potential benefit for patients and the reduction of wasteful duplication across healthcare sites as motivation for reporting QI work - **Environmental Context & Resources:** Heavy workload alleviated by allowing protected time for QI work, convening multidisciplinary writing teams, embedding local or regional QI research units that could operate in the same way as clinical trials units, providing structured programmes of QI education or mentorship and involving patients, who could also be part of a QI multidisciplinary team - **Skills:** Constraints imposed by word counts could be alleviated by uploading supplementary material and podcasts, - **Social influences**: encouraging multiple publications for a single QI study, - **Environmental Context & Resources**: using web-enabled formats that allow the reader to explore topics in more depth depending on what they are most interested in, and sections dedicated to negative studies in journals.   Barriers:   - **Environmental Context & Resources:** The broad scope of QI reporting – ranging from small local projects to multisite research across different disciplines – causes uncertainty about where QI work should be published - **Skills:** Challenges of reporting active ingredients and contexts in QI. Context is fundamental to the success of a QI intervention but difficult to report in ways that support replication and development - **Environmental Context & Resources**: Proximal challenges, such as the challenge of doing QI work and writing it up, while simultaneously looking after patients. - **Knowledge**: practical challenges such as restrictive word counts and reporting guidelines might have only a limited role in improving the quality of reporting, especially if QI stakeholders do not realise they exist - **Environmental Context & Resources**: distal, structural influences (such as norms about the format and content of biomedical research reporting), leading to incomplete reporting of QI findings - **Beliefs about consequences**: explaining failure may be so difficult that negative or null QI studies may never be written up or published. |
| Lim et al (2014) | **Original** | **Descriptive survey design**  A 13-question, online survey to assess medical oncologists attitudes toward and involvement in QI and perceived barriers to publishing QI studies. | **Understanding barriers and facilitators**  This paper examined the prevailing attitudes of medical oncologists toward QI and causes for the low QI publication rate in the medical oncology literature. | **Medical oncologists**  **Canadian Association of Medical Oncologists (CAMO) organisation**  **Canada**  143 oncologists responded out of the 332 surveys sent to members of the Canadian Association of Medical Oncologists (CAMO) from 52 different institutions across eight of 10 provinces in Canada. | Barriers   - **Knowledge**: unfamiliarity with QI methodology - **Social/Professional Role and Identity**: nonacademic focus - **Environmental Context & Resources:** lack of time - **Knowledge:** no identifiable journals for publishing QI studies - **Beliefs about Consequences**: perception that QI initiatives were of local institutional interest only - **Beliefs about Consequences**: Perception that small-scale projects were not suitable as academic contributions. |
| Matulis, Manning (2023) | **Non-original** | **Commentary**  Perspective focused on supporting success in QI rigor, credibility, spread, and publication. The author presents a case study of two QI teams that had differing approaches and varying degrees of success. The authors then reflect on the pros and cons to each approach. | **Understanding barriers and facilitators**  In this perspective article, we offer QI leaders practical suggestions to identify challenges in publishing QI and strategies to overcome those challenges | **Multidisciplinary groups led by an internal medicine physician and resident**  **Setting not defined**  **USA**  Two multidisciplinary groups led by an internal medicine physician and resident | Facilitators (org):   - **Goals**: Early, realistic assessment of scholarly ambitions. - **Environmental Context & Resources**: careful review of resources and support needed and provide a catalog of potential funding opportunities and resources. - **Goals:** Team building, authorship discussion, workload distribution - **Knowledge:** Introduce team to the SQUIRE guidelines and to identify venues for publication. - **Goals**: early consideration of the study of intervention - **Goals**: Manuscript preparation formalised as part of project work – toll-gate process to assure concurrent scholarly progression, accountability and timelines developed. - **Environmental Context & Resources:** Provide resources supporting QI publication – connect teams to available funding resources, target resources to support data analysis, IRB navigation and publication progress - **Environmental Context & Resources**: develop a small grant/fund to support QI manuscript preparation time - **Environmental Context & Resources**: Official sanctioning and support of a QI community forum by the organization will send the message that this work is both important and valued.   Barriers:   - **Goals**: Inexplicit initial assessment of publication goals. Frontline QI teams often do not sufficiently plan for the dissemination of their work, including publication in a peer-reviewed journal, at the start of their project. - **Behavioural regulation**: Teams start writing too late. Challenges arise when teams do not begin preparing their manuscript until after they have completed their QI project. - **Environmental Context & Resources:** Difficulty finding time. Inadequate time for performing regular work of the QI project and additional work necessary for publication. - **Environmental Context & Resources:** Funding limited for those interested in completing and publishing QI work. - **Environmental Context & Resources:** Difficulty finding data expertise and mentorship. Finding expertise and support in data collection and analysis - **Environmental Context & Resources:** Data availability. Publication may require data that are difficult to obtain without institutional electronic health record support. Often, teams may depend on manual data collection, or quality assurance data that is not be adequately rigorous for publication in journals. - **Environmental Context & Resources:** Other – navigating the IRB and determining whether a review is required - **Knowledge:** finding journals that understand and publish QI. - **Knowledge:** Unfamiliarity with elements of QI writing, including communication of subtle contextual elements, the application of formal and informal frameworks for understanding system performance, and QI study designs |
| Mormer, Stevans (2019) | **Non-Original** | **Commentary**  Compilation of library database search, author expertise, and first-hand experience. Definitions of QI activities explained and an overview of models and methods utilized in the implementation of QI programs provided. Examples of useful tools and QI projects in speech-language pathology and audiology were included. Benefits and opportunities, and barriers and facilitators were addressed. | **Understanding barriers and facilitators**  This article is intended to serve as a tutorial for speech-language pathology and audiology clinicians and researchers interested in pursuing QI practice and research | **Speech-language pathology and audiology clinicians**  **Setting not defined**  Targeted at speech-language pathology and audiology clinicians interested in quality improvement (QI)  practice and research | Facilitators   - **Beliefs about consequences:** Thoughtful and objective language used in conducting and reporting QI projects in order to overcome resistance – explanation of goals and findings to staff - **Environmental Context & Resources:** SQUIRE guidelines – recognition of legitimacy of QI research   Barriers   - **Beliefs about consequences –** QI may not be perceived as having the same value as traditional HSR articles - **Beliefs about consequences** Individual resistance to QI can occur as a project is launched, during implementation, or upon completion. - **Emotion:** QI endeavours have the potential to cause pressure or anxiety for those personnel involved in the unit under study - **Skills**: Lack of QI skills training in health professions curriculum - **Environmental Context & Resources**: lack of faculty who can teach QI competencies, learning QI on the job - **Environmental Context & Resources**: May need IRB approval for publication in journal, even if not required to conduct the study |
| Pearlman, Swanson (2021) | **Non-Original** | **Commentary**  The final instalment of a series of invited publications on topics related to QI in neonatal-perinatal medicine.  This paper provides a practical guide to publishing a QI paper – a review of some general concepts on how to optimally write and submit manuscripts for publication, as well as specific guidance regarding QI papers. | **Understanding barriers and facilitators**  This paper describes the best practices and common pitfalls when writing and publishing QI manuscripts in neonatal-perinatal medicine. Common pitfalls to avoid are also highlighted. | **Neonatal perinatal medicine**  **USA**  Target audience not explicitly defined | Facilitators:   - **Behavioural regulatio:** Writing and publishing should occur near the completion of the QI project, generally when sustained improvement has been achieved. Consider beginning to write the paper during the QI project with focus on introduction, context, interventions, measures to assess impact - **Goals**: Consider which journal is best: previous publishing of QI studies, population, specialty, target audience, impact factor, acceptance rate - **Social/professional role identity**: Authorship should be discussed early in the project, determined by one’s contribution to the design and implementation - **Environmental Context & Resources**: Follow formatting guidelines of the journal - **Environmental Context & Resources:** Use of SQUIRE guidelines for formatting QI study   Barriers:   - **Skills:** No definition of the local problem or context - **Goals**: Unidentified SMART goal - **Skills:** No description of QI methodology and tools - **Skills:** Using a protocol developed by others without adaptation - **Skills:** Lack run charts or process control charts to display continuous data - **Beliefs about capabilities:** Not discussing limitations - **Skills:** Implying causality |
| Schondelmeyer, Brower, Statile et al (2018) | **Non-original** | **Commentary**  A report as part of a series on important considerations in writing QI manuscripts, with a focus on how it differs from writing traditional clinical research reports.  This report introduces the SQUIRE guidelines as an important tool for writing and reviewing QI publications, and discusses the potential pitfalls and questions that arise when using the SQUIRE guidelines to write and publish QI work | **Understanding barriers and facilitators**  This report builds on the first 2 articles in the series to cover important considerations in writing quality improvement manuscripts with a focus on how it differs from writing traditional clinical research reports. | **Target audience and/or Setting not defined**  **USA** | Facilitators:   - **Environmental Context & Resources:** SQUIRE guidelines - **Goals:** Explicitly state the specific aim (measurable, actionable, relevant, time-bound) - **Skills:** Include the most relevant contextual factors (staffing, trainees, leadership, clinic set-up etc) - **Skills**: Provide a clear description of complex measures – measure definition, rationale, validity, reliability, Avoid jargon - **Skills:** Provide a clear, detailed description of each plan-do-study-act cycle, address reliability in the manuscript (the number of actions that achieve the intended result over the total number of actions taken), esp for a project that succeeded but did not reach its goal - **Beliefs about consequences:** Provide a clear rationale of why the intervention(s) would lead to improved outcomes - **Skills:** Use well-annotated run charts and statistical process control charts - **Goals:** Describe plans for sustainability and spread |
| Simon, Starmer, Conway et al (2012) | **Non-original** | **Commentary**  Review of the literature & experience with the Pediatric Research in Inpatient Settings (PRIS) network in QI & with QI projects | **Understanding barriers and facilitators**  We address the importance, current state, accomplishments, and challenges of QI and QI research in pediatric hospital medicine; define  the role of the PRIS Network in QI research; describe an exemplary QI research project, the I-PASS Study; address challenges for funding, training and mentorship, and publication; and identify future directions for QI research in pediatric hospital medicine | **Paediatric hospitalists**  **Setting not defined**  **USA** | Facilitators:   - **Environmental Context & Resources:** access to federal and non-federal sources of funding sources to both produce evidence and implement it into practice - **Environmental Context & Resources & Reinforcement:** The improvement-focused line of scientific inquiry and service should be rewarded and nurtured through training, mentorship, and promotions - **Environmental Context & Resources:** support the development of new fellowship programs with scholarly content and to foster additional training opportunities in QI and QI research methodology. - **Environmental Context & Resources:** Use of SQUIRE guidelines - **Skills:** Attention to methodological rigor in QI will allow the hospitalists to conduct QI that leads to meaningful and sustained changes to the system of health care. - **Environmental Context & Resources**: Specific guidance on QI could help pediatric hospitalists improve their projects, as well as the resulting publications. - **Environmental Context & Resources**: academic medical centers and community hospitals will need to create infrastructure to support faculty and staff interested in QI and implementation science. The improvement-focused line of scientific inquiry and service should be rewarded and nurtured through training, mentorship, and promotions   Barriers:   - **Knowledge**: Definition of QI vs QI research - **Environmental Context & Resources:** Limited funding for QI - **Skills**: Lack of doctors trained in QI conduct and lack of experience in writing a manuscript - **Environmental Context & Resources**: Difficulty finding mentors for QI due to junior hospitalists - **Reinforcement**: Lack of reward structure for those doing QI/research, standard promotions paradigms may not adequately reward their scholarly and academic work - **Environmental Context & Resources:** Traditional publication guidelines have not provided sufficient guidance on the design, types of interventions, data collection, and analysis for QI or QI research - **Skills:** Many hospitalists who are writing projects for publication do not have formal training in manuscript preparation |
| Van Cleave, Dougherty, Perrin (2011) | **Original** | **Qualitative Study**  Review and summary of a workshop conducted at the Pediatric Academic Societies 2007 meeting in Toronto, Canada, on conducting and publishing QI research.  Interviewed experts about common reasons QI research fails to reach publication  Reviewed recently published (2001-2009) paediatric QI articles to find specific examples of tactics to enhance publishability, as identified in interviews and the workshop | **Understanding barriers and facilitators**  To identify barriers to publishing results of paediatric QI research and provide practical strategies that QI researchers can use to enhance publishability of their work | **Journal editors, QI experts, QI & health services researchers, administrators**  **Paediatric Academic Societies meeting**  **Canada**  Workshop presenters: editors of 2 paediatric journal and other QI experts  Workshop attendees: not explicitly defined  Interviewees (7 total): QI researchers, administrators, journal editors, health services researchers who have reviewed QI manuscripts | Facilitators:   - **Social influences**: Study team; collaboration with researchers, participation in research network - **Environmental Context & Resources:** institutional commitment to QI scholarship - **Environmental Context & Resources:** Better research training for QI experts - **Knowledge:** Defining the question: identify and select a quality problem with gaps in knowledge of effective interventions, apply an effective QI method to a new population - **Skills:** Designing the intervention: base intervention on empirical evidence from literature review, select interventions that could practically be implemented elsewhere - **Skills:** Sample and setting: characterise population with baseline demographic and practice-level data, test intervention in diverse group of patients and practice settings, determine and obtain sample size needed to detect appropriate differences in outcomes before conducting study - **Skills:** Study design: use control group (incl waitlist control group), randomly assign to intervention versus usual care, use interrupted time-series analysis or mixed method if control group not practical - **Environmental Context & Resources:** Measuring outcomes: obtain various perspectives (staff perceptions, patient clinical and satisfaction outcomes, process measures), be persistent and complete in data collection, use standardised measures and data-collection tools (e.g. validated survey instruments) - **Environmental Context & Resources:** Manuscript: follow SQUIRE guidelines for reporting QI studies   Barriers:   - **Skills**: QI experts lack expertise in research methods - **Reinforcement**: Lack of incentive and support for publishing findings - **Knowledge:** Defining the question: quality problem and interventions are too well studied and do not represent ‘new knowledge’ - **Skills:** Designing the intervention: intervention does not have good face validity - **Environmental Context & Resources:** Sample and setting: intervention tested in a non-generalisable population or setting, sample too small - **Skills:** Study design: not robust, study underpowered to detect significant effects - **Skills:** Measure outcomes: measures too few, irrelevant or inaccurate - **Skills**: Manuscript: writing is unclear, intervention not well described |
| Wagstaff, Moonesinghe (2023) | **Non-original** | **Commentary**  Editorial in British Journal of Anaesthesia following decision to include QI publications in this journal  This editorial summarises the current problems with conducting, evaluating and publishing QI studies. It highlights existing guidance for prospective authors to follow regarding the reporting of QI interventions, their context(s), underlying theories, and evaluation. | **Understanding barriers and facilitators**  We hope to encourage the publication of more QI studies of sufficient quality to facilitate learning or replication elsewhere | **Audience/participants not defined**  **Anaesthesia**  **United Kingdom** | Facilitators:   - **Environmental Context & Resources:** Template for Intervention Description and Replication (TIDieR) checklist – guide to describing interventions in sufficient detail to be replicated by others - **Environmental Context & Resources:** use of SQUIRE guidelines - **Skills:** Description of context is crucial - **Skills:** Include consideration of theory (how and why intervention might cause predicted impacts) - **Beliefs about consequences:** Holistic appreciation of impacts, including those which are unanticipated, applies to the wider system or represents opportunity costs of engaging with the intervention - **Skills:** Include explicit descriptions of evaluative and analytical techniques, including use of qualitative techniques alongside quantitative ones - **Environmental Context & Resources:** Utilise national QI programmes/resources and tools to aid with design, implementation, evaluation and reporting of QI   Barriers:   - **Environmental Context & Resources:** Inconsistent, vague and variable application of terminology across disciplines - **Skills**: Poor quality and completeness in reporting QI - **Environmental Context & Resources:** QI projects often don’t fit norms of traditional biomedical academic publishing, as they are heterogenous, using broad scopes of interventions and methodologies – challenge to standardise the reporting of adaptive and iterative projects - **Environmental Context & Resources:** Limited time to write up studies alongside clinical responsibilities. - **Beliefs about consequences:** Reporting bias – positive studies more likely to be reported - **Skills:** Crucial details often omitted in intervention, QI method, context, unintended consequences – inhibits replication |

| 1. **STUDIES THAT ADDRESSED BARRIERS & FACILITATORS** | | | | | |
| --- | --- | --- | --- | --- | --- |
| **Author; Year** | **Study Type** | **Methodology** | **Aim** | **Participants & Setting** | **Outcomes** |
| Carpenter, Nieva, Albaghal, et al (2005) | **Original** | **QI tool development**  Development of The Dissemination Planning Tool for the Agency for Healthcare Research and Quality (AHRQ); Developing the tool began with adapting Rogers’ seminal diffusion theory. Literature was reviewed from health care, sociology, organizational development, psychology, and social sciences. Tools currently used in field-specific instances were reviewed. Sources were synthesized through a process of refinement, expert review, and testing. | **Addressing barriers and facilitators**  The Dissemination Planning Tool was developed to assist the AHRQ Patient Safety grantees with disseminating their research results. | **Researchers/experts in patient safety, dissemination, knowledge management**  **AHRQ**  **USA**  Expert reviewers: experienced health services researchers in PS research, national and international experts in dissemination research, professors in dissemination theory, knowledge management professionals, leaders in research dissemination organisations, professionals responsible for developing and maintaining dissemination partnerships  Patient safety researchers were recruited to complete the draft tool with their own research in mind. | Findings:   - **Environmental Context & Resources:** ‘This tool is an important contribution; there generally appears to be an absence of practical dissemination planning tools for researchers, and it is nice to see this need addressed.’ Receptivity to this planning tool and the noted dearth of similar tools reinforce the need to convert dissemination theory into practical tools and techniques - **Knowledge:** ‘I learned a great deal completing it’ - **Knowledge** ‘It provides explicit and detailed thinking’ ‘I found myself iterating my thinking as I went through each question – a very effective developmental tool.’ - **Social Influences**: ‘I plan to assemble the research team to gather additional input’ - **Beliefs about consequences**: Researchers traditionally have not been expected to think about dissemination of research results for use in practice. Researchers will benefit by understanding the dissemination process, recognising the importance of research’s use and its practical application - **Environmental Context & Resources**: It provides a structure to think about what can appear to be a nebulous charge to which researchers are increasingly expected to respond - **Knowledge:** The DPT is designed to promote awareness among researchers about where their research might and should be applied in practice |
| Cooley, Nelson, Slack et al (2015) | **Original** | **Intervention study – Curriculum implementation**  A multi-faceted intervention to increase student scholarly output was developed that included; 1) 120 minute lecture, 2) Abstract workshops, 3) Poster workshops, 4) A reminder at an advanced pharmacy practice experience meeting encouraging students to publish or present posters.  The intervention effect was measured as the percentage of students who presented at meetings and the number of student projects published, compared to a pre-intervention student class group. | **Addressing barriers and facilitators**  To increase the percentage of state, national, or international student presentations and publications | **Pharmacy students**  **Pharmacy department of one university**  **USA**  94 students from University of Arizona College of Pharmacy (compared to 95 students in year prior to intervention) | Findings:   - **Behavioural regulation:** this curricular intervention increased QI scholarly output   - A significant increase in the percent of students who presented posters or published manuscripts after the intervention (64% vs 81%)   - Total student productivity increased from 84 to 147 posters, publications and presentations   - The number of projects presented or published increased from 50 to 77 in one year |
| Henchey, Keefe, Munger et al (2020) | **Original** | **Intervention study – Structured research program**  Mentored capstone projects. Project proposals were solicited from faculty members and local colleagues, and students were matched with an individual project and mentor. After developing a written research proposal, students completed the project with mentor oversight, culminating with a poster session and completion of a manuscript. Students' knowledge of biostatistics, research confidence, and attitudes regarding research were evaluated using a validated survey instrument. Students and mentors were surveyed for feedback, and students' publications and presentations were tracked. | **Addressing barriers and facilitators**  To assess the impact of a Doctor of Pharmacy (PharmD) capstone project on students’ ability to conduct research and quality improvement, and to assess the feasibility of requiring projects in the core curriculum | **Doctor of Pharmacy students**  **One university**  **USA**  62 Third and fourth year Doctor of Pharmacy students at the University of Utah College of Pharmacy & 43 mentors working within or outside of the university | Findings:   - **Environmental Context & Resources:** Mentorship capstone programme with research/QI projects - **Knowledge:** There were no significant changes in students’ knowledge - **Intentions:** There were no significant changes in students’ attitudes, however, students’ interest in learning more about biostatistics decreased slightly - **Skills:** the number agreeing that they understand the statistics that they encounter significantly increased. - **Beliefs about capabilities:** There was a significant increase in students’ confidence scores, p,.001.Students’ confidence in their ability to understand and participate in research increased - **Beliefs about consequences:** 73% felt their project would make them more competitive for a job or residency - **Reinforcement**: Further, 88% of mentors reported that they were somewhat or extremely satisfied with the final project produced, 73% thought the students’ work was beneficial to the mentor, 97% thought the project was a valuable learning experience for their student, and 79% felt they could write a strong letter of recommendation for their student based on their project mentoring experience. - **Environmental Context & Resources**: Most mentors in both years reported spending “a reasonable amount of time” on each activity related to mentoring, ranging from 63% to 92%, depending on the year and activity. - **Behavioural regulation:** A structured research programme yielded completion and dissemination of projects. 58% of students presented posters at national conferences. 21% published manuscripts in peer-reviewed journals. - **Behavioural regulation:** Of these students, 61 (97%) completed their projects on schedule by April 2019. Two students fell behind and joined the following class of students. |
| Kooker, Latimer, Mark (2015) | **Original** | **Intervention Study – Structured Research Programme**  Structured programme involving 6 months of evidence-based practice writing workshops with a writing coach to support nurses to publish a manuscript on a previously-completed EBP project. During the workshops, the coach delivered a curriculum on writing a manuscript. Their manuscripts were evaluated throughout by the coach and workshop faculty, with feedback provided. Each session had a Read Aloud activity for the team to share feedback and make clarifications. A tracking tool was designed to monitor the teams’ progress. In the final workshop, the programme was evaluated by the participants. | **Addressing barriers and facilitators**  This article describes the structured approach, timeline, writing activities, and coaching guidance used to support the publication of 12 QI articles in one nursing journal | **Bedside nurses**  **Setting not defined**  **USA**  Bedside nurses participated. There were 13 teams, with multiple authors for each paper. | Findings:   - **Environmental Context & Resources**: Writing group meeting monthly with an external writing coach - **Social influences**: Peer support, including gentle peer pressure, was also seen as a positive aspect. Several participants also voiced their appreciation of timely coaching and support from workshop faculty. - **Environmental Context & Resources**: Logistics of sessions were facilitated: different facilities for access, parking, presentation/logistical support, promotion of small group communication. Alternating session times. Access to food/snacks. - **Environmental Context & Resources**: Clear link between the manuscript topical outline and the structure of the Iowa Model was made to ease the transition to the manuscript format. - **Goals**: Having a defined submission date. Setting an expectation that teams would meet the external deadlines imposed by the journal editor. - **Social influences:** Commitment to a person known and respected by the group – lead guest editor of the journal was present at the first workshop - **Skills**: writing team members need technical assistance with drafting and editing skills - **Behavioural Regulation:** 82% of the assignments were completed on time. Knowing that final submission of each paper was needed by a set date to meet the publication date encouraged author adherence to interim deadlines. - **Behavioural Regulation :** Thirteen manuscripts were submitted for the final editorial review, and 12 were accepted for publication - **Environmental Context & Resources**: dedicated time during the workshop needs to be set aside for actual writing by the team members. - **Knowledge:** One intervention not adequately evidence-based and therefore rejected by the journal – need more rigorous selection of projects for future workshops |
| McNab, McKay, Bowie (2015) | **Original** | **Intervention Study – Structured research programme**  Pilot study – QI training package developed and delivered to core medical trainees and GP trainees, involving 1-day workshop and mentoring during completion of a  QI project over 3 months.  Mixed methods evaluation with data collection via questionnaire surveys, knowledge assessment, formative assessment of project proposals, completed QI projects and publication success.  Quantitative analysis of survey responses, thematic analysis of one-to-one communications. | **Addressing barriers and facilitators**  **Conducting and disseminating QI/PS research**  We aimed to design and deliver a quality improvement training package to core medical and general practice specialty trainees, and evaluate impact in terms of project participation, completion and publication in a healthcare journal | **Core medical trainees, GP trainees**  **Scotland NHS board areas**  **United Kingdom**  20 total trainees submitted QI proposals: 11 core medical trainees and 12 GP trainees in the five west of Scotland NHS board areas  Three withdrew following training day – lack of time due to other career priorities (23 trainees completed training) | Findings:   - **Environmental Resources and context:** 1-day workshop and mentoring during completion of a QI project - **Knowledge**: Training/education programme in QI, esp explanation of the QI tools, open discussion of previous Qi projects including critical analysis of a published report, group exercises on developing appropriate improvement measures. Knowledge and confidence in all aspects of QI improved during the pilot. Although an increase in QIKAT score was observed (46% vs 54%) it was not statistically significant (P=0.17). - **Skills**: Majority agreed that training had been effective in teaching them how to perform QI project and changed how they would perform QI in the future (10/14) - **Belief about consequences**: Confidence in aspects of QI knowledge and skills was very low overall, improved after training and was largely maintained at four months. - **Social influences**: Having a mentor when undertaking the project was helpful. Initial feedback from mentor on QI proposal in planning the project. - **Environmental Context & Resources**: Eight participants found using BMJ quality as a resource was useful - **Behavioural regulation**: Twenty participants submitted a project proposal (87%). Ten completed quality improvement projects (43%), eight were judged as satisfactory (35%), and four were submitted and accepted for journal publication (17%). - **Environmental Context & Resources**: Lack of time due to other career/educational priorities - **Goals**: Poor preparation and planning at initial project design stage - **Social influences**: Limited project buy-in from colleagues |
