# Supplementary material for: Barriers and facilitators to disseminating quality improvement and patient safety research: a scoping review
Source: Int J Qual Health Care. 2025 Aug 28;37(3):mzaf084. doi: 10.1093/intqhc/mzaf084 (PMC12459987; doi:10.1093/intqhc/mzaf084)
Supplement: mzaf084_Supplementary_Data [file mzaf084_supplementary_data.zip › Supplementary_material_1.docx]

**Supplementary Material 1: Search strategy for Medline (Ovid)**

1. exp quality improvement/
2. (quality adj1 improvement).ti,ab.
3. QI.ti,ab.
4. exp patient safety/
5. (patient adj1 safety).ti,ab.
6. (quality adj1 improvement adj3 research).ti,ab.
7. (patient adj1 safety adj3 research).ti,ab.
8. (quality adj1 improvement adj3 project*).ti,ab.
9. 1 or 2 or 3 or 4 or 5 or 6 or 7 or 8
10. hurdle*.ti,ab.
11. obstruct*.ti,ab.
12. discourag*.ti,ab.
13. challeng*.ti,ab.
14. facilitat*.ti,ab.
15. enabl*.ti,ab.
16. barrier*.ti,ab.
17. imped*.ti,ab.
18. concern*.ti,ab.
19. caus*.ti,ab.
20. advantag*.ti,ab.
21. disadvantag*.ti,ab.
22. burden*.ti,ab.
23. benefi*.ti,ab.
24. positiv*.ti,ab.
25. negativ*.ti,ab.
26. Issue*.ti,ab.
27. Problem*.ti,ab.
28. Strateg*.ti,ab.
29. Complex*.ti,ab.
30. 10 or 11 or 12 or 13 or 14 or 15 or 16 or 17 or 18 or 19 or 20 or 21 or 22 or 23 or 24 or 25 or 26 or 27 or 28 or 29
31. (conduct* adj3 research).ti,ab.
32. (conduct adj3 trial*1).ti,ab.
33. (professional adj3 (publish* or publication*1)).ti,ab.
34. (research adj3 (publish* or publication*1)).ti,ab.
35. (scholarly adj3 (publish* or publication*1)).ti,ab.
36. (scientific adj3 (publish* or publication*1)).ti,ab.
37. (academic adj3 (publish* or publication*1)).ti,ab.
38. (professional adj3 present*).ti,ab.
39. (research adj3 present*).ti,ab.
40. (scholarly adj3 present*).ti,ab.
41. (scientific adj3 present*).ti,ab.
42. (academic adj3 present*).ti,ab.
43. (submit* adj3 research).ti,ab.
44. (submit* adj3 journal*).ti,ab.
45. (disseminat* adj3 research).ti,ab.
46. (disseminat* adj3 quality adj1 improvement).ti,ab.
47. (disseminat* adj3 patient adj1 safety).ti,ab.
48. (research adj3 present*).ti,ab.
49. (conference adj3 present*).ti,ab.
50. (conference adj3 disseminat*).ti,ab.
51. (quality adj1 improvement adj3 (publish* or publication*1)).ti,ab.
52. (patient adj1 safety adj3 (publish* or publication*1)).ti,ab.
53. (patient adj1 safety adj1 research).ti,ab.
54. (quality adj1 improvement adj1 research).ti,ab.
55. ((QI) adj1 (publish* or publication*1)).ti,ab.
56. 31 or 32 or 33 or 34 or 35 or 36 or 37 or 38 or 39 or 40 or 41 or 42 or 43 or 44 or 45 or 46 or 47 or 48 or 49 or 50 or 51 or 52 or 53 or 54
57. 9 and 30 and 55
58. Limit 56 to (english language and yr="2001 -Current")
